# Targeting Peptidylarginine Deiminases in Neurons and Astrocytes in Central Nervous System Injury—Effects of Pan-PAD Inhibitor Cl-Amidine in an Oxygen–Glucose Deprivation Model of Ischaemia (OGD/R) and LPS Stimulation In Vitro

**DOI:** 10.3390/ijms27115118

**Published:** 2026-06-05

**Authors:** Dina Ahmed, Stephen J. Getting, Maria Ashioti, Sigrun Lange

**Affiliations:** School of Life Sciences, College of Liberal Arts and Sciences, University of Westminster, 115 New Cavendish Street, London W1W 6UW, UK; d.ahmed1@westminster.ac.uk (D.A.); s.getting@westminster.ac.uk (S.J.G.); m.ashioti@westminster.ac.uk (M.A.)

**Keywords:** peptidylarginine deiminases (PADs), deimination/citrullination, central nervous system (CNS), neuron, astrocyte, hypoxia, ischaemia, oxygen–glucose deprivation, Cl-amidine, regeneration

## Abstract

Peptidylarginine deiminases (PADs) are a family of five isozymes (PAD1–4, PAD6) in humans, with PAD2, 3 and 4 associated with the central nervous system. PAD-mediated post-translational citrullination/deimination of target proteins contributes to pathobiological processes, including in the central nervous system (CNS), where the potential of PAD inhibitor treatment has been reported. This study aimed to identify PAD-dependent pro-regenerative responses in neuronal and astrocytic cells, respectively, using human cellular in vitro models to assess the therapeutic effects of pan-PAD, PAD2- and PAD4 isozyme-specific inhibitors in an oxygen–glucose deprivation/reperfusion model of ischaemia (OGD/R) at different time windows (30 min, 1 h and 4 h) in conjunction with scratch injury and LPS stimulation. Key findings suggest that pan-PAD inhibitor Cl-amidine promotes CNS regeneration through enhancing wound-healing of both neuronal and astrocytic cells, indicating roles for several PAD isozymes in acute CNS injury. Astrocyte cells showed the most prominent PAD4 detection, with significantly lower levels of PAD1, PAD2, PAD3 and PAD6, while differentiated SH-SY5Y neuronal cells showed the highest detection of PAD3, followed by PAD2 and PAD1, as well as strong PAD6 positivity, but negligible PAD4 detection. Histone H3 citrullination was significantly reduced in response to Cl-amidine treatment in both cell types, indicating changes in histone H3-dependent events in CNS injury. Cl-amidine treatment modulated key neuronal (beta-3 tubulin) and astrocytic (GFAP) markers and also reduced inflammatory cytokine IL-6 levels in astrocytes following 4 h OGD/R in conjunction with LPS stimulation. This study indicates roles for several PAD isozymes, with differing prominence in neurons and astrocytes, and emphasises the potential for pharmacological PAD inhibitor treatment in CNS injury.

## 1. Introduction

Acute central nervous system (CNS) injuries include cerebral ischaemia (stroke) and traumatic brain injury (TBI). These represent leading causes of global mortalities and debilitation, with TBI affecting almost 69 million people and stroke affecting above 15 million people annually [1]. Other debilitating forms of acute CNS injury include hypoxic-ischemic encephalopathy (HIE), which affects 1–3 per 1000 neonates [2], and spinal cord injury (SCI), which affects over 15 million people globally [3].

There is increasing evidence for critical roles of peptidylarginine deiminases (PADs) within the context of CNS pathologies, including acute CNS injuries, neurodegenerative disease and CNS-associated cancers [4,5,6,7,8,9,10,11,12,13,14,15,16,17,18,19,20]. PADs constitute a family of five calcium dependent isozymes in humans, PAD1, PAD2, PAD3, PAD4 and PAD6, which can convert arginine into citrulline in proteins, causing post-translational deimination/citrullination. Deimination renders the polar arginine residues of target proteins into neutral citrulline, affecting conformation and function of target proteins, generation of neoepitopes which contribute to inflammatory responses and effects on epigenetic regulation via citrullination of histones, including histone H3 (mainly via PAD2 and PAD4), which is also implicated in extracellular trap formation which can in some instances contribute to exacerbated injury responses [21,22,23].

Various models of acute CNS injuries have been used to assess roles for neuronal and astrocytic responses [24,25,26,27,28,29]. Studies in HIE and TBI models have revealed that protein deimination affects multiple brain regions [5,10], and similar observations have been reported in neurodegenerative diseases [16,30]. Pharmacological PAD inhibition, particularly using the pan-PAD inhibitor Cl-amidine, has shown promise in several in vivo animal models of acute CNS injury [4,5,31]. As translation from preclinical in vivo rodent models remains a challenge [32]; further dissection of PAD-mediated mechanisms and the potential for pharmacological PAD inhibitors in human CNS injury in vitro systems are of interest to further current understanding of the different PAD isozymes in neuronal and astrocytic responses.

This study utilised immortalised human neuronal (Retinoic-Acid differentiated SH-SY5Y) and astrocytic (SVG-P12) cells to assess PAD isozymes and protective effects of PAD inhibitors using an in vitro model of oxygen–glucose deprivation model of ischaemia (oxygen–glucose deprivation with reperfusion; OGD/R) in conjunction with scratch injury. The scratch injury model, which was utilised in this current study, is generally considered a well-established in vitro form of mechanical trauma [24,25,28,33] assessing cellular migration and gap closure as a function of wound-healing capacity. OGD/R can mimic cerebral ischaemia in vitro by combining glucose and serum-deprivation of the cell cultures while simultaneously incubating them under hypoxic conditions (0.1 or 1% oxygen) [34], while application of *Escherichia coli* lipopolysaccharide (LPS) is a commonly used agent to mimic neuroinflammation [35]. The oxygen/glucose deprivation model mimics the interruption of blood flow and metabolic deficiency as a result of reduced oxidative metabolism, leading to the downstream pathways of cerebral ischaemia in neurons. The addition of reperfusion initiates the reperfusion-injury cascade as a result of reoxygenation, which occurs in the majority of clinical stroke cases and causes exacerbation of the disease process [36]. This is the most clinically translatable and most extensively utilised model of ischaemic stroke in cellular stroke research [37]. The combination of mechanical injury, OGD/R and endotoxin mimic the pathophysiological downstream pathways and inflammatory, apoptotic and necrotic processes that occur as a result of traumatic brain injury resulting in secondary brain injury from cerebral ischaemia [38].

The current study aimed at assessing wound-healing (pro-regenerative) effects of pharmacological PAD inhibition in neuronal and astrocytic cells in OGD/R in vitro, also in the presence of LPS stimulation, with a focus on the pan-PAD inhibitor Cl-amidine [4,5,39,40,41], in addition to PAD2 inhibitor AMF-30a [42] and PAD4 inhibitor GSK-199 [43], as studies on these PAD isozyme-specific inhibitors in CNS regeneration are lacking.

We hypothesise that PAD inhibitor treatment promotes wound-healing and anti-inflammatory responses, with differing effects on neuronal and astrocytic cells. Effects on gap closure speed following scratch injury were assessed alongside changes in histone H3 citrullination as a specific citrullination target, indicative of epigenetic regulation and associated with neuroinflammatory responses [4,5,44]. In the neurons, changes in beta-3-tubulin (neuronal differentiation) and nestin (stemness marker) were assessed, while in the astrocytic cells, changes in Glial Fibrillary Acidic Protein (GFAP) and S100B were evaluated, as these play roles in the astroglial response [45,46,47,48]. Changes in the release of pro-inflammatory cytokines Interleukin-1β (IL-1β) and Interleukin-6 (IL-6) were also assessed in both cell types as indicative of neuroinflammatory modulation [49].

## 2. Results

### 2.1. PAD Isozyme Detection Differs in Neuronal and Astrocytic Cells

**Figure 1:**
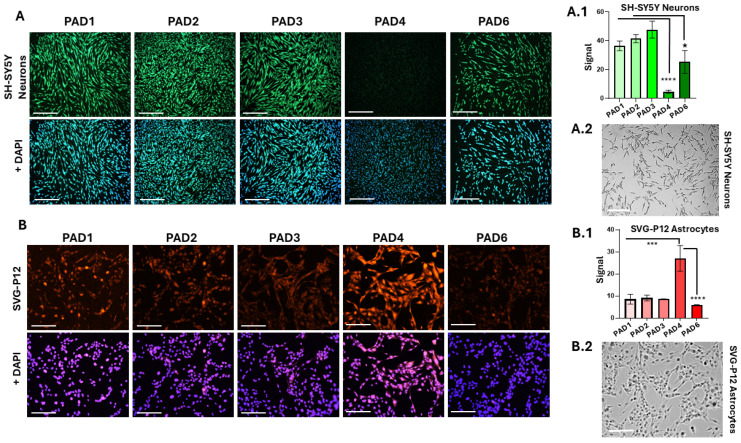
PAD isozyme detection in neurons and astrocytes in vitro. (**A**,**B**) Detection of PAD isozymes (PADs 1–4 and PAD6) by ICC comparing (**A**) neuronal (differentiated SH-SY5Y) and (**B**) astrocytic (SVG-P12) cells, with nuclear DAPI co-staining (blue) shown in parallel. Strong neuronal detection of PADs 1, 2 and 3 was observed, while moderate astrocytic detection was observed, localised both to the cytoplasm and nuclei. PAD4 levels were high in astrocytes, both in cytoplasm and nuclei, but negligible in the neurons. PAD6 showed strong detection with nuclear localisation in the neurons, while exhibiting low levels in the astrocytes. (**A.1**,**B.1**) Quantification of positive signal detection by ICC for the five PAD isozymes in neurons (**A.1**) and astrocytes (**B.1**) (n = 3 biological replicates; * *p* < 0.05; *** *p* < 0.001; **** *p* < 0.0001). (**A.2**,**B.2**) Brightfield images confirm the morphology of differentiated SH-SY5Y cells (neurons; (**A.2**)) and SVG-P12 (astrocytes; (**B.2**)) cells. The images were taken using the EVOS™ FL Auto 2 Imaging System (AMAFD2000) and the 10× objective (scale bar = 275 µm).

Figure 1 shows PAD isozyme detection on differentiated SH-SY5Y cells (Figure 1A) and SVG-P12 cells (Figure 1B) using anti-PAD antibodies against all five human-expressed PAD isozymes, with quantification of immunodetection for each isozyme presented for neuronal cells (Figure 1(A.1)) and astrocyte cells (Figure 1(B.1)) and the morphology of both cell lines shown in Figure 1(A.2,B.2).

The neuronal (differentiated SH-SY5Y) cells showed strongest positive staining for PAD1, PAD2 and PAD3—detectable both in cytoplasm and nucleus, which aligns with their previously reported positive expression within the human CNS [15]. In the neuronal cells, levels of PAD4 were negligible, while PAD6 detection showed nuclear staining as confirmed by co-localisation with DAPI (nuclear blue, fluorescent stain) (Figure 1A).

In the SVG-P12 cells, PAD 1 showed similar levels to PAD2 and PAD3, exhibiting moderate cytoplasmic and nuclear detection. PAD4 showed the significantly strongest positive detection levels, aligning with roles for astrocytes in immunity, whilst PAD6 detection was lowest (Figure 1B).

### 2.2. Pharmacological PAD-Inhibition Affects Scratch Injury Closure in Neuronal and Astrocytic Cells Under Normoxic Conditions

Differentiated SH-SY5Y cells at a uniform, confluent, monocellular layer were exposed to scratch injury followed by application of either 100 µM pan-PAD inhibitor Cl-amidine, 10 µM of PAD2 isozyme-specific (AMF-30a) or 5 µM of PAD4 isozyme-specific (GSK-199) inhibitors, to assess effects on pan-PAD (including PAD3) inhibition and PAD2 or PAD4 isozyme-specific inhibition, respectively, based on doses from the published literature [4,18,39,42,43].

In all scratch injury experiments, the gap measurement (volume) was assessed at 0 h, 24 h and 48 h post-scratch to identify the timepoint to use for measurement of significant changes in wound closure in further experiments due to effects of PAD inhibitor application versus control scratch.

**Figure 2:**
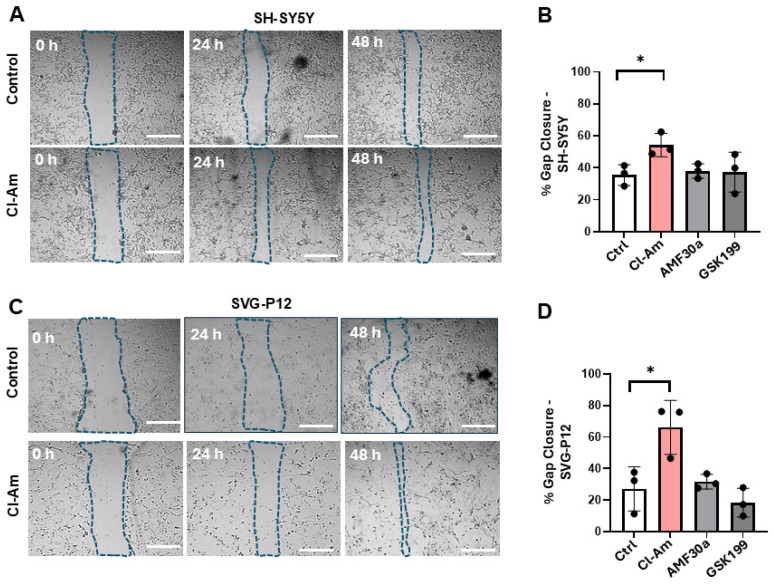
Effects of PAD inhibitors on neuronal and astrocyte cells scratch injured under normoxic incubation with serum and glucose-rich media. (**A**) Representative images of wells showing scratch injury (highlighted with the dashed outline) of control untreated versus Cl-amidine-treated neuronal cells under normoxic conditions. Images were captured by EVOS using the 4× objective; scale bar indicates 625 µm. (**B**) Bar graph comparing gap closure in control cells not receiving PAD inhibitor versus Cl-amidine, AMF30a (PAD2 inhibitor) and GSK199 (PAD4 inhibitor)-treated neuronal cells, with significant gap closure observed at the 48 h timepoint (*t*-test, n = 3; * *p* ≤ 0.05) for Cl-amidine. (**C**) Representative images of wells showing scratch injury (highlighted with the dashed outline) of control untreated versus Cl-amidine-treated astrocytes. Images captured by EVOS using the 4× objective; scale bar indicates 625 µm. (**D**) Bar graph comparing gap closure in control cells not receiving PAD inhibitor versus Cl-amidine, AMF30a- and GSK199-treated astrocytes, with significant gap closure observed at 48 h post-scratch for Cl-amidine (n = 3 technical replicates; unpaired *t*-test comparing control versus respective Cl-am treatment, * *p* ≤ 0.05).

Cl-amidine significantly promoted gap closure of the differentiated neurons at 48 h post-scratch (Figure 2A,B; n = 3), while wound closure was not statistically significant at the 24 h timepoint (Figure 2A). There was no significant change observed for gap closure in response to application of the PAD2 (AMF30a) and PAD4 (GSK199) isozyme-specific inhibitors in the neuronal cells (Figure 2B). Assessment of scratch injury in the SVG-P12 cells, showed that gap closure was also significantly increased by Cl-amidine at 48 h post-scratch (Figure 2C,D), but not at the 24 h timepoint (Figure 2C). Significant effects of the PAD2 and PAD4 isozyme-specific inhibitors were not observed on gap closure of the astrocytic cells (Figure 2D). While both cell lines showed similar profiles for gap closure, Cl-amidine accelerated gap closure in astrocytes more than in neurons, which may mirror the higher proliferative potential of astrocytes.

### 2.3. Effects of Pan-PAD-Inhibitor on Scratch Injury of Neuronal Cells and Astrocytes Following OGD/R and LPS Stimulation

Based on the wound-healing effects observed in the scratch injury model with pan-PAD inhibitor Cl-amidine under normoxia, but negligible effects of the PAD2 (AMF30a) and PAD4 (GSK199) isozyme-specific inhibitors, as well as previous studies showing neuroprotective effects for Cl-amidine in acute CNS injury animal models [4,5,31], the effects of Cl-amidine in the context of OGD/R and LPS stimulation were next assessed, using the same in vitro models of neurons and astrocytes, respectively.

**Figure 3:**
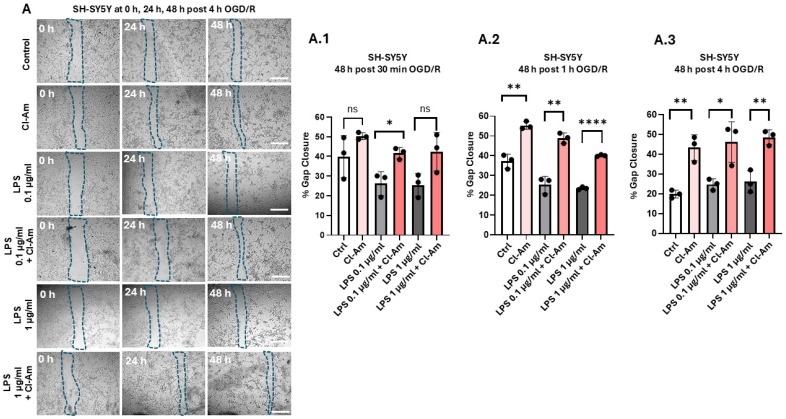

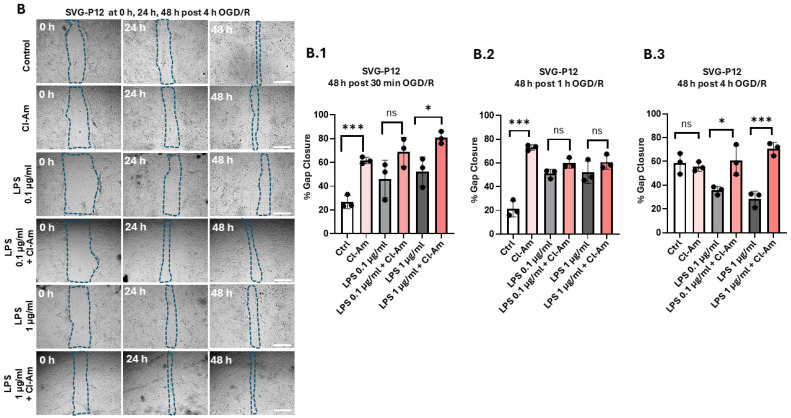
Effects of Cl-amidine on gap closure in the OGD/R scratch injury of neuronal and astrocyte cells exposed to 30 min, 1 h or 4 h hypoxia and LPS stimulation. Scratch assay was carried out in conjunction with exposure to 4 h hypoxia incubation with glucose–serum deprivation (OGD/R) with/without co-infection of either 0.1 or 1 µg/mL LPS and with/without Cl-amidine treatment (100 µM). (**A**) Representative images of scratch injury (highlighted with the dashed outline) gap closure in neuronal (differentiated SH-SY5Y) cells showing Cl-amidine-treated versus control cells not receiving PAD inhibitor or LPS, but still exposed to OGD/R, for the different conditions (hypoxia, LPS + hypoxia) at 4 h post-scratch (images captured by EVOS using the 4× objective; scale bar indicates 625 µm). (**A.1**–**A.3**) Bar graphs represent scratch injury results for neuronal cells exposed to 30 min, 1 h or 4 h hypoxic incubation with glucose–serum deprivation (OGD/R) with/without application of 0.1 or 1 µg/mL LPS and with/without Cl-amidine (100 µM). Ctrl = control cells exposed only to the OGD/R condition (n = 3 technical replicates, mean with SD; unpaired *t*-test comparing control versus respective Cl-am treatment, * *p* ≤ 0.05, ** *p* ≤ 0.01, **** *p* ≤ 0.0001). (**B**) Representative images of gap closure in astrocytes showing Cl-amidine-treated versus control cells (only exposed to the OGD/R condition) for the different OGD/R conditions (hypoxia, LPS + hypoxia) at 4 h post-scratch (images captured by EVOS using the 4× objective; scale bar indicates 625 µm). (**B.1**–**B.3**) Bar graphs representing scratch injury results for astrocytes exposed to 30 min, 1 h or 4 h hypoxic incubation with glucose–serum deprivation with/without co-infection with 0.1 or 1 µg/mL LPS and with/without Cl-amidine (100 µM). Ctrl = control cells exposed only to the OGD/R condition (n = 3 technical replicates, mean with SD; unpaired *t*-test comparing control versus respective Cl-am treatment, * *p* ≤ 0.05, *** *p* ≤ 0.001).

Differentiated SH-SY5Y cells were exposed to 30 min, 1 h or 4 h of hypoxic incubation with serum–glucose deprivation in combination with scratch injury to recapitulate OGD/R acute CNS injury (Figure 3).

The scratch injury was furthermore carried out in combination with either 0.1 µg/mL or 1 µg/mL LPS to mimic infection. All the experiments were terminated at 48 h post-injury, at which point the gap closure analysis was conducted, based on optimisation of the assay, showing at this point significant and measurable differences between control (OGD/R only) and treated (Cl-amidine and/or LPS) cells under most of the conditions (Figure 3).

Cl-amidine-treated neurons showed significantly faster gap closure under all stress conditions assessed, compared to cells in control (OGD/R only) wells, in 1 h and 4 h OGD/R conditions (Figure 3A—representative images are shown). Wound-healing (gap closure) effects of Cl-amidine were observed both for OGD/R also in the absence and presence of LPS treatment, with more marked effects seen in the cells receiving the higher LPS dose (1 µg/mL) in the 1 h and 4 h OGD/R conditions (Figure 3(A.2,A.3)). In the milder 30 min OGD/R model, Cl-amidine exhibited significant gap closure when combined with the lower 0.1 µg/mL LPS dose, and a trend showing increased gap closure overall (Figure 3(A.1)). In the 1 h OGD/R model, Cl-amidine promoted gap closure of neuronal cells compared with the control non-treated cells (OGD/R only) (Figure 3(A.2)). This was also observed in combination with LPS treatment, with significance reached for both LPS doses at 48 h post-scratch injury (Figure 3(A.2)). When using the 30 min OGD/R model, the neuroprotective effects of Cl-amidine were observed as significant when combined with the lower LPS dose (0.1 µg/mL) at 48 h post-insult (Figure 3(A.1)).

SVG-P12 cells were exposed to the same conditions as the differentiated neuronal cells and assessed for changes in gap closure following scratch injury (Figure 3B,(B.1–B.3)). Cl-amidine treatment significantly promoted gap closure in the 4 h OGD/R model in combination with LPS stimulation at both doses (Figure 3B,(B.3)). Wound-healing (gap closure) effects of Cl-amidine on the astrocytic cells were also observed in the shorter 30 min (Figure 3(B.1) and 1 h (Figure 3(B.2)) OGD/R models, although less pronounced effects were observed for the LPS stimulated wells for those timepoints (Figure 3(B.1–B.3)).

### 2.4. Pan-PAD Inhibitor Cl-Amidine Reduces Histone H3 Citrullination (CitH3) in Neuronal and Astrocyte Cells in OGD/R

**Figure 4:**
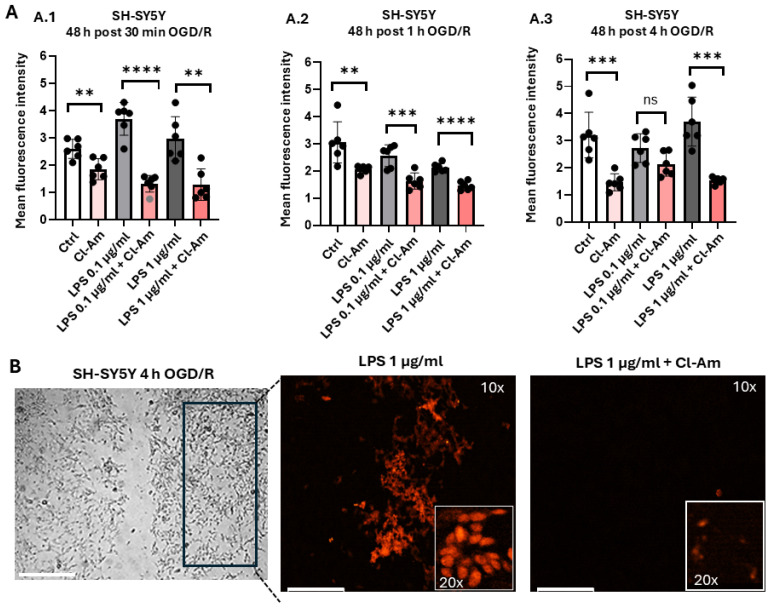

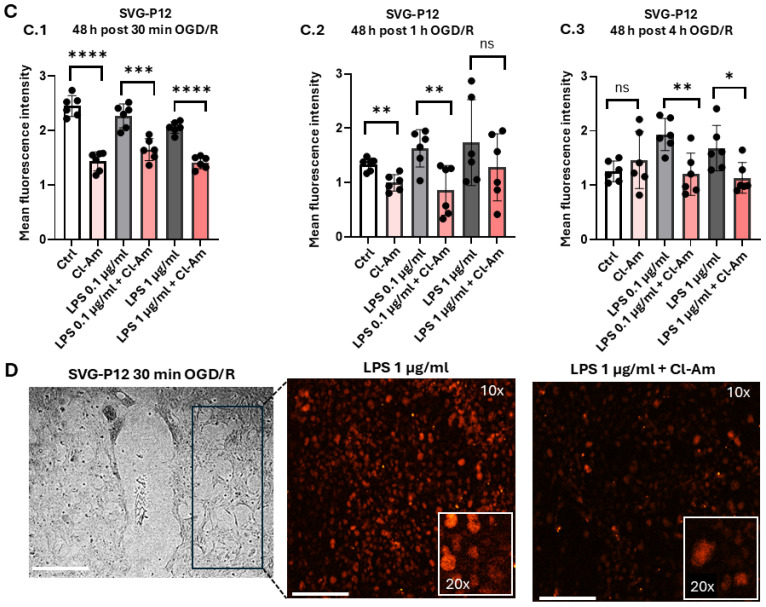
Pan-PAD inhibitor Cl-amidine reduces CitH3 in neuronal and astrocyte cells in most conditions OGD/R and in conjunction with LPS stimulation. (**A**) Bar charts comparing CitH3 levels as detected by ICC in neuronal cells treated with Cl-amidine versus cells not receiving Cl-amidine, at all three OGD/R timepoints: (**A.1**) 30 min OGD/R; (**A.2**) 1 h OGD/R; (**A.3**) 4 h OGD/R. CitH3 staining was measured by mean fluorescence intensity (EVOS captured images with the 10× lens, RFP channel). The values were compared between cells receiving Cl-amidine versus cells not receiving Cl-amidine, within each experimental group (n = 6, mean with SD; unpaired *t*-test, ** *p* ≤ 0.01, *** *p* ≤ 0.001, **** *p* ≤ 0.0001). (**B**) Representative images are shown for Cl-amidine (100 µM)-treated neuronal cells for the 4 h OGD/R model in the presence of LPS stimulation (1 µg/mL), showing reduced CitH3 levels by ICC (Alexa Fluor red stain) compared to cells not receiving Cl-amidine (images captured using the 10× objective, adjacent to the scratch injury, with inserted boxes showing higher magnification images at 20×; captured using EVOS; scale bar indicates 275 µm). (**C**) Bar charts comparing CitH3 levels as detected by ICC in astrocytes treated with Cl-amidine versus cells not receiving Cl-amidine, at all three OGD/R timepoints: (**C.1**) 30 min OGD/R; (**C.2**) 1 h OGD/R; (**C.3**) 4 h OGD/R. CitH3 staining was measured by mean fluorescence intensity (EVOS captured images with the 10× lens, RFP channel). The values were compared between cells receiving Cl-amidine versus cells not receiving Cl-amidine, within each experimental group (n = 6 technical replicates, mean with SD; * *p* ≤ 0.05; ** *p* ≤ 0.01; *** *p* ≤ 0.001; **** *p* ≤ 0.0001; unpaired *t*-test comparing control versus respective Cl-am treatment). (**D**) Representative images are shown for effects of Cl-amidine treatment on CitH3 levels in astrocyte (SVG-P12) cells in the 30 min OGD/R model in the presence of LPS stimulation (1 µg/mL), showing reduced CitH3 staining in the Cl-amidine-treated cells (images captured with the 10× objective on EVOS; inserted boxes show higher magnification images at 20×; scale bar indicates 275 µm). In all graphs, Ctrl = control cells exposed only to the OGD/R condition.

Protective effects of Cl-amidine to reduce histone H3 citrullination (CitH3) as part of promoting neuronal and astrocytic wound-healing were assessed by immunocytochemistry (ICC) in the OGD/R model for the three different timepoints (30 min, 1 h and 4 h) (Figure 4). In the neurons for 30 min OGD/R, Cl-amidine significantly reduced CitH3 levels compared with cells in control wells (OGD/R only) not receiving Cl-amidine, and significantly reduced CitH3 levels in the presence of both LPS doses assessed (Figure 4(A.1)). In the neurons for 1 h OGD/R, CitH3 was similarly reduced in all conditions (Figure 4(A.2)). In the neurons for 4 h OGD/R, significant reduction in CitH3 was observed in the OGD/R-treated cells receiving Cl-amidine, compared with the control wells (OGD/R only) where cells did not receive the PAD inhibitor, and for Cl-amidine treatment with the higher LPS dose (Figure 4(A.3)). Representative images of CitH3 detection in neuronal cells are presented for the 4 h OGD/R model, comparing cells receiving Cl-amidine in conjunction with LPS at the higher dose (1 µg/mL) compared with LPS stimulation alone (Figure 4B).

In the astrocytes for 30 min OGD/R, Cl-amidine significantly reduced CitH3 levels compared with cells in control wells (OGD/R only) not receiving Cl-amidine, and significantly reduced CitH3 levels in the presence of both LPS doses assessed (Figure 4(C.1)), similar to what was observed in the neurons. In the astrocytes for 1 h OGD/R, CitH3 was significantly reduced in response to Cl-amidine treatment in the absence of LPS, and in the presence of the lower LPS dose, but did not reach statistically significant difference for the higher LPS dose (Figure 4(C.2)). In the astrocytes for 4 h OGD/R, significant reduction in CitH3 detection was observed in response to Cl-amidine treatment for both LPS doses tested (Figure 4(C.3)). Representative images of CitH3 detection in astrocytes are presented for the 30 min OGD/R model comparing cells receiving Cl-amidine in conjunction with LPS at the higher dose (1 µg/mL) compared with LPS stimulation alone (Figure 4D).

### 2.5. Pan-PAD Inhibitor Modulates Neuronal (β-3-Tubulin), Stemness (Nestin) and Astrocytic (GFAP and S100B) Markers in OGD/R

**Figure 5:**
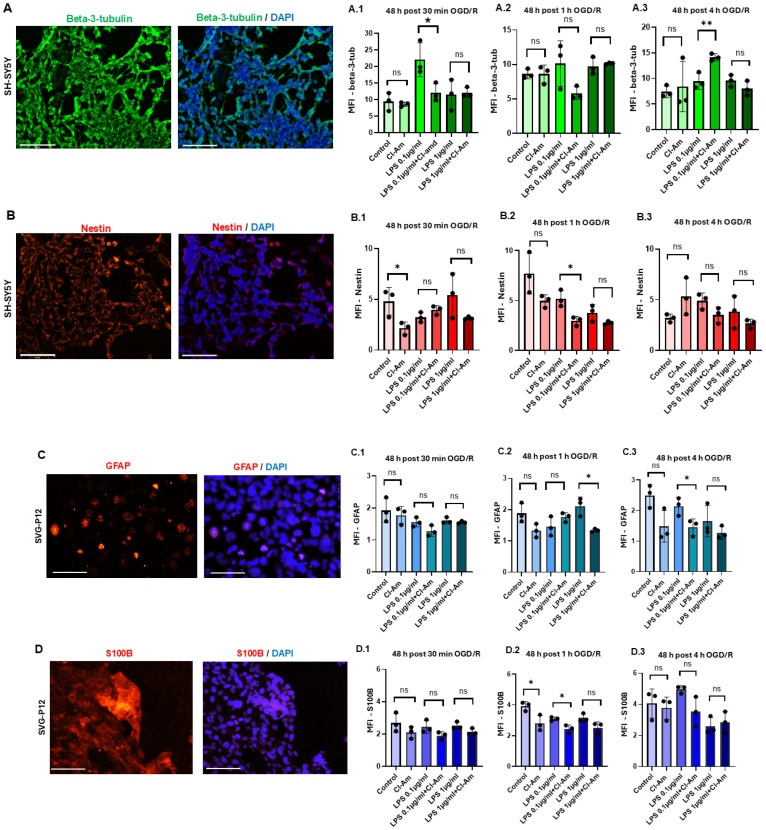
Assessment of neuronal (Beta-3 tubulin) and stem (nestin) markers in scratch injured neuronal cells, astrocytic markers (GFAP and S100B) in SVG-P12 cells in OGD/R, and effects of Pan-PAD inhibitor Cl-amidine. (**A**) Beta-3 tubulin-positive staining is strongly detected in neuronal (differentiated SH-SY5Y) cells; examples shown from scratch injured cells exposed to 30 min OGD/R with 0.1 µg/mL LPS stimulation. (**A.1**–**A.3**) Bar charts showing the quantitative analysis (mean fluorescence intensity, MFI) of Beta-3 tubulin staining levels in all conditions at 48 h post 30 min OGD/R (**A.1**), 1 h OGD/R (**A.2**) and 4 h OGD/R (**A.3**) (n = 3 technical replicates, mean with SD; unpaired *t*-test comparing Cl-amidine treatment versus omitting the PAD inhibitor per condition, respectively, * *p* ≤ 0.05, ** *p* ≤ 0.01). (**B**) Nestin detection in differentiated SH-SY5Y cells, indicative of some progenitor-like cells in response to the injury; example shown at 1 h OGD/R + LPS 0.1 μg/mL. (**B.1**–**B.3**) Bar charts showing the quantitative analysis (mean fluorescence intensity, MFI) of nestin staining levels in all conditions at 48 h post 30 min OGD/R (**B.1**), 1 h OGD/R (**B.2**) and 4 h OGD/R (**B.3**) (n = 3 technical replicates, mean with SD; unpaired *t*-test comparing Cl-amidine treatment versus omitting the PAD inhibitor per condition, respectively, * *p* ≤ 0.05). (**C**) GFAP-positive staining in SVG-P12 astrocytic cells 48 h post 4 h OGD/R with 0.1 µg/mL LPS co-infection; some nuclear localisation of GFAP (red) is detected. (**C.1**–**C.3**) Bar charts showing the quantitative analysis (mean fluorescence intensity, MFI) of GFAP staining levels in all conditions at 48 h post 30 min OGD/R (**C.1**), 1 h OGD/R (**C.2**) and 4 h OGD/R (**C.3**) (n = 3 technical replicates, mean with SD; unpaired *t*-test comparing Cl-amidine treatment versus omitting the PAD inhibitor per condition, respectively, * *p* ≤ 0.05). (**D**) S100B detection is shown in SVG-P12 cells scratch injured and exposed to 1 h OGD/R 0.1 µg/mL LPS, at 48 h post-injury. (**D.1**–**D.3**) Bar charts showing the quantitative analysis (mean fluorescence intensity, MFI) of S100B staining levels in all conditions at 48 h post 30 min OGD/R (**D.1**), 1 h OGD/R (**D.2**) and 4 h OGD/R (**D.3**) (n = 3 technical replicates, mean with SD; unpaired *t*-test comparing Cl-amidine treatment versus omitting the PAD inhibitor per condition, respectively, * *p* ≤ 0.05). All representative images also include the merged images with DAPI and were captured using EVOS using the 20× objective, with scale bars = 125 µm. In all graphs, Ctrl = control cells exposed only to the OGD/R condition.

The results showed that under all conditions, the differentiated SH-SY5Y cells stained strongly for beta-3 tubulin (Figure 5A), confirming their neuronal properties. The changes in staining intensities of beta-3-tubulin were assessed and quantified under all OGD/R conditions of the scratch injury model at 30 min, 1 h and 4 h, as well as in response to LPS stimulation and Cl-amidine treatment (Figure 5(A.1–A.3)). In the shorter 30 min model, beta-3 tubulin levels were significantly increased in response to 0.1 µg/mL LPS and reduced in response to Cl-amidine treatment (Figure 5(A.1)). In the 1 h OGD/R model, a trend was observed for reduced beta-3 tubulin levels in the Cl-amidine-treated OGD/R + LPS (0.1 µg/mL) neuronal cells, compared with the LPS stimulation alone (Figure 5(A.3)). In the 4 h OGD/R model, beta-3 tubulin was elevated in response to Cl-amidine treatment in the LPS-treated (0.1 µg/mL) neuronal cells (Figure 5(A.3)).

Nestin detection was assessed in the scratched OGD/R neuronal cells as an indicator of changes in potential stemness properties, which could be part of a pro-regenerative response, and showed some dynamic changes, although interpretation requires caution (Figure 5B). Nestin levels were significantly decreased in response to Cl-amidine treatment in the 30 min OGD/R model, with a similar trend observed in combination with the higher LPS dose (1 µg/mL), albeit not statistically significant (Figure 5(B.1)). In the 1 h OGD/R model, Cl-amidine significantly decreased nestin detection in LPS-treated (0.1 µg/mL) neuronal cells (Figure 5(B.2)), while no significant effects were observed in the other conditions. No significant changes were observed for nestin levels in the 4 h OGD/R experiment (Figure 5(B.3)).

In the SVG-P12 astrocyte cells, two canonical astrocytic markers, GFAP and S100B, were assessed by ICC under all experimental conditions compared with controls (Figure 5C,D). GFAP detection showed an overlay with the nuclear DAPI staining in the SVG-P12 cells under all conditions (representative image in Figure 5C). Changes in GFAP levels were not statistically significant between treatment groups in the 30 min OGD/R experiment, while there was an observed trend of overall decrease in GFAP-positive staining in the Cl-amidine-treated groups (Figure 5(C.1)). In the 1 h OGD/R, experiment a statistically significant decrease in GFAP staining was observed for the Cl-amidine-treated group in combination with the higher LPS dose, compared with the LPS treatment alone (Figure 5(C.2)). In the 4 h OGD/R experiment, a significant decrease in GFAP staining was observed in the Cl-amidine-treated group in combination with the lower dose of LPS, compared with the LPS treatment alone (Figure 5(C.3)). When assessing S100B-positive staining (Figure 5D), Cl-amidine showed a trend (albeit non-significant) of reducing S100B-positive signal in all conditions assessed (Figure 5(D.1–D.3)), with significant effects observed in the 1 h OGD/R experiment (*p* ≤ 0.05), also in conjunction with LPS stimulation at the lower dose (* *p* ≤ 0.05) (Figure 5(D.2)).

### 2.6. Effects of Pan-PAD Inhibitor Cl-Amidine on Pro-Inflammatory Cytokines IL-1β and IL-6 in Neurons and Astrocytes

**Figure 6:**
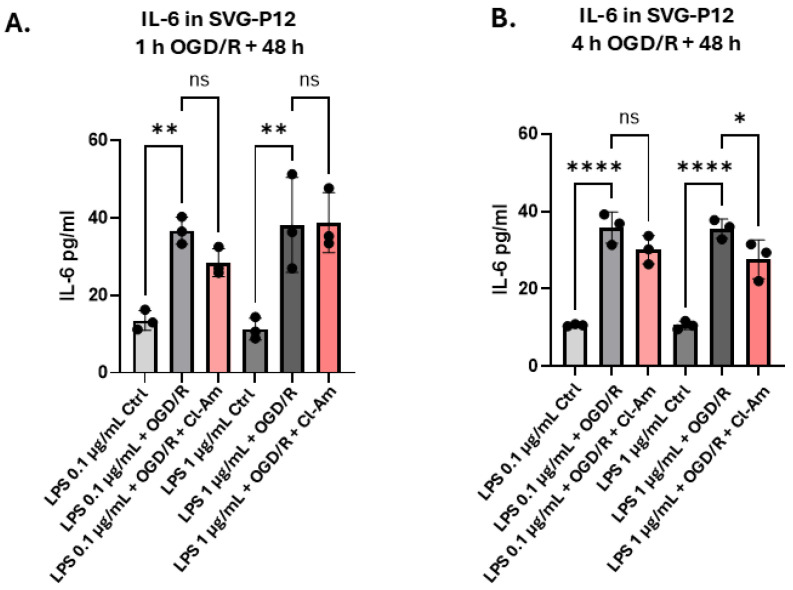
IL-6 detection in SVG-P12 astrocytes using ELISA. (**A**) SVG-P12 cells were exposed to 1 h OGD/R, and IL-6 levels measured 48 h post-insult. Control wells received LPS (0.1 µg/mL LPS or 1 µg/mL LPS) without OGD/R; treated wells received OGD/R and LPS (0.1 µg/mL LPS or 1 µg/mL LPS) in the presence or absence of Cl-Am (100 µM). (**B**) SVG-P12 cells were exposed to 4 h OGD/R in combination with LPS stimulation (0.1 µg/mL LPS or 1 µg/mL LPS) alone or in combination with Cl-amidine (100 mM). Control wells were not exposed to OGD/R and only treated with LPS. IL-6 levels were determined by ELISA 48 h post-insult. Data are represented as mean ± SD (n = 3 technical replicates; one-way ANOVA, * *p* ≤ 0.05; ** *p* ≤ 0.01; **** *p* ≤ 0.0001).

Effects of Cl-amidine treatment (100 µM) on changes in IL-1β and IL-6 cytokine levels in the neuronal (differentiated SH-SY5Y) and astrocyte (SVG-P12) cells were evaluated by ELISA test at 48 h post 30 min, 1 h and 4 h OGD/R, with and without LPS stimulation (0.1 or 1 μg/mL). Results showed negligible changes in both cytokines in the neuronal cells under all conditions tested. In the SVG-P12 cells, no changes were observed for IL-1β, while some differences were observed in the 1 h and 4 h OGD/R groups for IL-6, as presented in Figure 6. In 1 h OGD/R (Figure 6A), LPS in conjunction with OGD/R increased IL-6 levels significantly by approximate 2.5-fold, compared with LPS alone in control wells, but Cl-amidine addition did not significantly reduce IL-6 levels in this experimental setup (Figure 6A). In the 4 h OGD/R model, a significant increase (~3.5- to 5-fold) of IL-6 levels was observed for LPS in combination with OGD/R, compared with LPS alone in control wells, and this was significantly reduced in response to Cl-amidine treatment in the cells receiving the higher LPS dose (1 μg/mL) (approximately 22%; * *p* < 0.05; Figure 6B).

## 3. Discussion

This study assessed roles for PADs in acute CNS injury of OGD/R and neuroinflammation, highlighting the use of in vitro modelling for PADs in human neuronal and astrocyte cell cultures, respectively. The SH-SY5Y neuroblastoma immortalised cell line was differentiated into neurons using retinoic acid (RA) and has been utilised in various in vitro studies of TBI and ischaemia [50,51,52,53,54,55,56]; hence, it was the neuronal in vitro model of choice for this current study. The astrocyte SVG-P12 immortalised cell line has been used for studying ischaemia [57] and as a control in glioblastoma studies [58,59], providing the rationale for its use in our current study. Differences in PAD isozyme prominence were detected in the two cell lines, with the differentiated SH-SY5Y neuronal cells showing the highest levels of PAD3, followed by PAD2 and PAD1, but negligible levels of PAD4. In the astrocytes, PAD4 detection was dominant, followed by significantly lower levels of PAD1, PAD2 and PAD3. The detection of several PADs in both cell lines correlates with neuroinflammatory roles for astrocytes in the CNS mediated by PAD2 and PAD4, including via modulation of histone citrullination in relation to extracellular trap formation and possible epigenetic effects in neurons, as indicated by strong CitH3 staining, which can be related to previous findings in animal models, showing elevated CitH3 levels in acute CNS injury which were reduced by Cl-amidine treatment [4,5,60]. An unexpected finding was the strong positive detection of PAD6 in the neuronal cells, while PAD6 levels in astrocytes were much lower, as this isozyme is mainly associated with developmental processes [61,62], but aligns with recent reports of PAD6-positive staining in human post-mortem brain samples [15].

The pan-PAD inhibitor Cl-amidine was more effective than the PAD2 and PAD4 isozyme-specific inhibitors to promote gap closure in both cell lines, with the most marked effects in the 4 h OGD/R insult model. The potential of pharmacologically targeting several PADs to reduce CNS injury does align with the detection of several PADs in both cell lines and correlates with previous in vivo models of spinal cord injury, with HIE and TBI showing neuroprotective and pro-regenerative effects for Cl-amidine [4,5,31,63].

In addition to OGD/R, LPS was used to mimic inflammatory responses, and both LPS doses tested (0.1 and 1 µg/mL) correlated with increased levels of CitH3 staining. In macrophages, LPS has been shown to activate PADs and induce CitH3 production in vitro [64]. Histone H3 citrullination (including by PAD2 and PAD4) is a key player in neuroinflammatory responses and has been linked to NETosis [44,65,66,67], while NETosis inhibition has, furthermore, been shown to improve the overall clinical outcomes post-injury and ischemia [31,68]. Here, Cl-amidine treatment significantly decreased CitH3 levels in both neuronal and astrocytic cells, under most conditions assessed. This correlates to previously reported findings in several in vivo studies assessing CitH3 staining and NETosis including in spinal cord injury, HIE and TBI [4,5,60,69,70]. As antibodies specific to citrullinated targets are scarce, CitH3 has been a citrullination target most frequently assessed as a readout of PAD-mediated events, particularly relating to PAD2 and PAD4. Immunostaining observed in this study by ICC does correlate with staining patterns observed in CNS in vivo models, both using fluorescence and chromogenic detection [4,5,14,15].

Some changes in neuronal differentiation (beta-3 tubulin) and stemness (nestin) markers, as well as in astroglial (GFAP and S100B) markers, were observed in response to OGD/R and following Cl-amidine treatment.

Beta-3 tubulin constitutes the dynamic end of the cytoskeletal microtubules [71] and was used as a marker of neuronal differentiation for the SH-SY5Y cells. Cl-amidine treatment enhanced neuronal migration following scratch injury, as represented by increased speed of gap closure, in the 1 h and 4 h OGD/R model, with and without LPS. Cl-amidine treatment increased overall beta-3 tubulin levels, which aligns with possible roles in promoting neuronal migration [72,73]. Cytoskeletal remodelling via cytoskeletal protein citrullination may be modified, including beta-3 tubulin, which has been previously reported to change in response to PAD inhibitor treatment [71,74]. Our observed effects of Cl-amidine affecting beta-3 tubulin levels may be of considerable importance for CNS protection as the contribution of beta-3 tubulin to neurogenesis and axonal regrowth has been reported in various in vivo neuronal injury and knockout models [75,76,77]. PAD2-induced citrullination of beta-3 tubulin has, for example, been shown to be crucial for regulating cytoskeletal dynamics in murine gonadotropic cells [74]. Further investigations into beta-3 tubulin citrullination must be carried out in future studies.

Nestin, a class VI intermediate filament protein, was used as a marker for neural stemness [78,79,80,81] to assess possible changes in possible neuroprotective properties following OGD/R and in response to Cl-amidine treatment. Nestin is generally inversely proportional to the level of neuronal differentiation markers [82], and this was observed in the 30 min and 1 h OGD/R models, both with and without LPS, where an increase in beta-3 tubulin coincided with a decrease in nestin levels. The only exception, where nestin showed a trend for increased levels (but not statistically significant), was in the 4 h OGD/R model in Cl-amidine-treated cells compared with control OGD/R (absence of LPS). Given the vague results on nestin observed in our current study, additional markers indicative of pro-regenerative cellular responses, including Ki-67 as a proliferation marker in neurogenesis [83], could be assessed in addition.

Previous studies have reported elevated nestin levels in areas of prolonged hypoxic- ischemic insult and injury [84,85]. Nestin is associated with neurogenesis in ischaemic stroke models [86], and nestin-positive SVZ-derived neural stem/progenitor cells have been shown to migrate towards ischaemic areas [87]. As nestin is suggested to be a citrullination target similar to other intermediate filaments [88], it may be of interest to assess citrullination of nestin in future CNS injury studies.

In the glial cells, GFAP levels were significantly decreased in LPS-stimulated cells treated with Cl-amidine in the 1 h OGD/R and 4 h OGD/R models, indicating neuroprotective effects of PAD inhibition, as GFAP increase is associated with neuroinflammatory responses in TBI [89]. S100B levels showed cytoplasmic localisation and a trend for reduced levels in response to Cl-amidine treatment, but only significantly in the 1 h OGD/R model in the absence of LPS. S100B is a main contributor to neuronal survival and differentiation along with further numerous cellular activities, most importantly brain tissue repair upon extracellular secretion by the astrocytes [48]. Post brain injury, both GFAP and S100B are considered of clinical relevance, specifically in TBI [48,89,90], where the serum level of both proteins can be directly correlated to the extent of existing brain damage post-injury [90]. GFAP has been suggested as a blood biomarker in TBI, SCI and ischaemia [47,91,92]. While astrogliosis is considered beneficial for initial wound-healing, and thus considered neuroprotective, prolonged astrogliosis can impede neuronal regeneration. The results of our study may suggest that Cl-amidine could promote astrocytic gap closure partly viaCitH3-mediated events, alongside some modulation of GFAP, although this requires further investigation. GFAP is a known citrullination target, with neo-epitope formation contributing to gliosis events, and in a rodent retinal gliosis model, Cl-amidine was shown to reduce protein citrullination [93]. An interesting observation in the OGD/R model in our current study was a nuclear detection of GFAP, which warrants further investigation. A recent study described perinuclear aggregates formed by a de novo variant of GFAP-alpha isoform in primary astrocytopathy [94]. It may be possible that oxidative-stress-mediated events and post-translational modifications in the OGD/R model may affect GFAP intracellular distribution and may be of interest in future studies [95,96,97,98,99,100]. In relation to the presented findings, it must be noted that both the SH-SY5Y and SVG-P12 are immortalised and/or cancer-derived cell lines with proliferative capacity. Hence, the use of the wound-healing model by scratch injury as representative of regeneration may need further validation using a proliferation inhibitor and additional viability controls to distinguish directed cell migration from enhanced cell survival and ongoing proliferation. Effects on cytoskeletal, including actin, remodelling should also be further investigated. Some limitations for comparison of ICC staining between samples using mean fluorescence intensity (MFI) must also be acknowledged, as differences of number of cells in the field of view, microscope parameter settings for imaging and the ICC protocol may add variances. While other approaches for a more direct comparison of immunopositive cell numbers could also be carried out, MFI was the choice of measurement, choosing fields of view adjacent to the scratch injury with similar cell density across samples compared; images were taken using the same microscope parameters for all comparisons carried out, the same ICC protocol was applied for each experiment, and post-processing of images was uniform.

Assessment of the pro-inflammatory cytokines IL-1β and IL-6 by ELISA showed no changes in the neuronal cells, while in the astrocytes a significant increase was observed for IL-6 levels in the 4 h OGD/R model. Furthermore, Cl-amidine treatment significantly decreased elevated IL-6 levels in the presence of LPS (1 µg/mL) stimulation. Several studies using in vivo murine models have reported that IL-6 contributes to the selective activation of astrocytes that underlies their response to CNS injury [101,102,103,104,105]. Both in vivo and in vitro studies reported that PAD2 exhibits progressive overexpression in astrocytes undergoing astrogliosis [106,107,108] and Cl-amidine was shown to reduce IL-6 expression in myeloma alongside reducing CitH3 [109]. AS the SVG-P12 cells showed higher levels of PAD4 than PAD2, which coincided with significantly elevated IL-6 levels, a role for PAD4 in addition to PAD2 in IL-6 secretion may be suggested in these cells. Additional neuroinflammatory parameters such as TNF-α [110,111], should be assessed in future studies, as well as oxidative stress and hypoxia-related markers (such as HIF-1a) [112,113], to further dissect PAD-mediated mechanisms and protective effects of PAD inhibition in acute CNS injury. While the use of individual cell lines can provide some valuable insights into cell-specific properties, the physiological relevance of findings from in vitro models may be limited. Our findings do, though, overall align with published studies from various CNS injury in vivo models, which is reassuring. Considerable variability was observed for several experiments, which may be attributed to the small sample size (n = 3 or n = 6), and further validation in a larger sample size will, therefore, be needed.

Overall, the CNS protective effects of Cl-amidine observed in our study highlight its role as a potent pan-PAD inhibitor, with the ability to target several PAD isozymes depending on their dominance in the cell types.

## 4. Materials and Methods

### 4.1. Cell Culture and Neuronal Cell Differentiation

SH-SY5Y neuroblastoma cells (ATCC, CRL-2266) were initially cultured in T-75 flasks using complete growth medium comprising 1:1 DMEM/HAM-F12 medium containing GlutaMax (A4192001, Gibco, Fisher Scientific, Leicestershire, UK), 10% heat inactivated fetal bovine serum (FBS) (A5670501, Gibco), 1% penicillin–streptomycin (5000 U/ml) (15070063, Gibco) and 1% of non-essential amino acids (NEAA) (11140050, Gibco) in 95% air and 5% CO_2_. The medium was changed every 48 h until the cells reached 80% confluence. Thereafter, SH-SY5Y cells were trypsinised before proceeding to the neuronal differentiation protocol, using 10 µM retinoic acid (RA, all trans-retinoic acid; ATRA (R2625, Merck, Sigma-Aldrich, Gillingham, UK)) which was supplemented to the complete growth medium to generate a neuronal differentiation medium [114]. The SH-SY5Y cells were seeded at 25, 30, 40 and 60 × 10^3^ cells/well in Nunc Cell-Culture treated 24-well plates (142475, Fisher Scientific) to identify optimal seeding density, in the presence of the differentiation medium which was regularly changed every 48 h for a six-day period to achieve complete neuronal differentiation; this was confirmed with beta-3 tubulin staining (Supplementary Figure S1). Optimal seeding density was determined as 4 × 10^4^ cells/well, and used thereafter as this generated a uniform confluent monocellular layer of differentiated cells by day 6 (Supplementary Figure S1). Gentle manual swirling of the plates was carried out to achieve even distribution of the cells across the wells before incubation, to avoid cellular adherence and clustering at the peripheral sides of the walls.

SVG P12 astrocytic cells (ATCC, CRL-8621) were cultured in T-75 flasks using DMEM/F12complete growth medium containing GlutaMax (A4192101, Gibco), 10% heat inactivated FBS (A5670501, Gibco), 1% penicillin–streptomycin (5000 U/ml) (15070063, Gibco) and 1% NEAA (Gibco Fisher, UK) in 95% air and 5% CO_2_. The medium was changed every 24 to 48 h until cells reached 80% confluence. Cells were then trypsinised and seeded at a cell density of 5 × 10^4^ cells/well in Nunc Cell-Culture treated 24-well plates (142475 Fisher Scientific), according to published optimisation for scratch injury experiments [59]. Both cell lines were used at passages 6 to 8.

### 4.2. Scratch (Wound-Healing) Assay

Scratch assay was performed on the differentiated SH-SY5Y cells (RA was removed from the medium before the scratch injury to avoid interference with the treatment interventions) and the SVG-P12 cells, respectively, using a 200 µL pipette tip to scrape the confluent monolayer of cells longitudinally across the middle of the wells. The culture medium was changed immediately after the scratch, whereafter either complete medium (control wells, omitting PAD inhibitor) was added or complete medium containing 100 µM pan-PAD inhibitor Cl-amidine (Cayman, No. 10599, Ann Arbor, MI, USA), 10 µM PAD2 inhibitor AMF30a (CAY10723, Cayman Chemical) or 5 µM PAD4-inhibitor GSK199 (MedChemExpress, 1549811-53-1, South Brunswick Township, NJ, USA) for differentiated SH-SY5Y (neurons), where RA was removed before application, and SVG-P12 (astrocytes) cells, depending on the experiment conducted, as further described below. Doses of the PAD inhibitors were chosen according to previously published studies on properties and effectiveness of the different pharmacological PAD inhibitors in other models [4,5,18,39,40,42,43,115].

The cell migration of both cell lines following scratch injury was determined as percentage of gap closure at 24 h and 48 h (endpoint of the experiments), calculated using the following formula: (A0 − At)/A0 × 100; where A0 is the scratched area at time zero, and At is the scratched area at 24 h or 48 h, respectively.

### 4.3. OGD/R in Conjunction with Scratch Injury

To mimic the effects of acute CNS injury under ischaemic insult, with/without infection, the following assays were carried out in 24-well plates on both cell lines, proceeding with pan-PAD inhibitor Cl-amidine as this provided the most wound-healing effects of the scratch injury in both cell lines under normal conditions: The scratched differentiated SH-SY5Y and SVG-P12 cells were exposed to 30 min, 1 h or 4 h OGD alone or in combination with either 0.1 µg/mL or 1 µg/mL of LPS (from *E. coli* O111:B4, LPS25, Merck, Sigma Aldrich, Gillingham, UK; mimicking infection) followed by reperfusion for 48 h. The cells were simultaneously exposed to oxygen and FBS deprivation, using serum- and glucose-free medium (Glucose-free DMEM, 11520416, Gibco), while for hypoxia, the cells were incubated in a hypoxia chamber (Innova CO-48 incubator, New Brunswick Scientific Co. Inc., Edison, NJ, USA) adjusted at 0.1% O_2_, 5% CO_2_, balance N_2_ and 37 °C. Pan-PAD inhibitor Cl-amidine was used at 100 µM concentration (according to [115]) throughout the reperfusion window, using normoxic incubation conditions at 95% air, 5% CO_2_, and incubated at 37 °C with glucose- and serum-enriched media comprising 10% FBS and 17.5 mM of D-Glucose. This was for assessment of therapeutic outcomes of pan-PAD inhibition following the different time windows of applied insult, which may inform clinical relevance of PAD inhibitor application post-insult. Following reperfusion for 48 h, cells were fixed for immunocytochemical staining. Control wells were run within each experimental setup as follows. Under normoxia conditions the control wells received the scratch injury, but PAD inhibitor application was omitted. In the OGD/R experiments, the control wells received scratch and OGD/R, but LPS and Cl-amidine application were omitted. In addition, OGD/R experiments assessed the effects of LPS alone, Cl-amidine alone or Cl-amidine in combination with LPS.

### 4.4. Immunocytochemistry on Differentiated SH-SY5Y and SVG-P12 Cells

Both cell lines were seeded on 24-well plates at a cell density of 40,000 cells/well for differentiated SH-SY5Y cells and at 50,000 cells/well for SVG P12 cells, for immunocytochemical staining of the PAD isozymes (PADs 1–4 and PAD6). Once cells reached 70–80% confluence, the media was removed, and wells were rinsed with Phosphate Buffer Saline + 0.1% Tween 20 (PBS-T), 1 mL/well. Scratch injury experiments were, furthermore, stained for CitH3, beta-3 tubulin, nestin, GFAP and S100B as appropriate.

The cells were fixed with 4% paraformaldehyde in PBS (pH 7.4) for 10 min at room temperature. The cells were thereafter washed 3 × 5 min with ice-cold PBS. Cell permeabilisation was performed for 10 min with PBS containing 0.1% Triton X-100, before washing the cells again three times for 5 min with PBS.

Blocking was performed with 1% BSA and 22.52 mg/mL glycine in PBS-T (PBS + 0.1% Tween 20) for 30 min at room temperature, followed by washing for 3 × 5 min in PBS-T. Incubation with primary antibodies was carried out overnight at 4 °C with the following antibody dilutions in PBS-T containing 1% BSA: anti-human PAD1, PAD2, PAD3 and PAD4 (ab181762, ab50257, ab50246, ab50247, Abcam, Cambridge, UK; diluted 1/200), anti-human PAD6 (PA5-72059, Thermo Fisher Scientific, Waltham, MA, USA; diluted 1/100), CitH3 (ab5103, Abcam; diluted 1/200), Beta-3 tubulin (ab52623, Abcam, diluted 1/500), nestin (ab18102, Abcam, diluted 1/500), GFAP (ab68428, Abcam, diluted 1/500) and S100B (MA5-42438, Invitrogen-Thermo Fisher Scientific, Waltham, MA, USA, diluted 1/500) in 1% BSA in PBS-T overnight at 4 °C to make a 100 µL solution in total per well. The negative control wells omitted the primary antibody and were incubated with 1% BSA in PBS-T.

Following primary antibody incubation, the wells were washed for 3 × 5 min in PBS, before application of the secondary goat IgG anti-rabbit antibody (Alexa Fluor 488 Abcam ab150077/green or 594 Abcam ab150080/red), except for nestin, where goat anti-mouse IgG secondary antibody was used (Alexa Fluor 647 Abcam ab150115/red), diluted at 1/500 in 1% BSA in PBS-T for 1 h at RT in the dark, followed by counterstaining with DAPI nuclear stain (Sigma Aldrich, St. Louis, MO, USA, D9542) (1 µg/mL), for 1 min. Imaging of the cells post-immunostaining was carried out using the EVOS™ FL Auto 2 Imaging System (AMAFD2000, Invitrogen, Thermo Fisher, UK) with the GFP, RFP and DAPI EVOS fluorescent channels for green, red, and blue fluorophores. Corresponding bright-field images were captured. For the scratch injury experiments, three fields of view adjacent to the scratch (upper, middle, lower) were evaluated per well, using the same size of field for all comparisons. Light intensity, exposure and gain were kept constant between captures. In order to quantify the fluorescence signal intensity, images were imported into ImageJ open-source software for image analysis https://imagej.en.softonic.com/ (v 1.54k, accessed on 27 May 2026) where uniform post-processing for background subtraction was carried out. Thereafter, mean fluorescence intensity (MFI) was measured per image using the mean values representing the intensity of the fluorescence per image to perform statistical analysis comparing the different conditions versus relevant controls.

### 4.5. Assessment of PAD Inhibition on Inflammatory Cytokines IL-1β and IL-6 by ELISA

The differentiated SH-SY5Y neuronal cells and SVG-P12 astrocyte cells were exposed to the different experimental conditions and PAD inhibitor treatment with Cl-amidine, at different timepoints, as described in the sections above. Commercially available ELISA kits (DY201 and DY206, R&D Systems, Bio-Techne, Abingdon, UK) were used to assess potential changes in pro-inflammatory cytokines IL-1 β and IL-6 in the cell-free supernatants of the cell cultures under the different conditions following the manufacturer’s instructions and according to previously described methods [116,117]. The commercial ELISA kits show negligible (<1%) cross-reactivity with other cytokines and chemokines according to the manufacturer (R&D systems).

### 4.6. Statistical Analysis

All experiments were carried out for n = 3 or n = 6 technical replicates, and data are represented as means with the error bars showing standard deviation (SD). Student’s unpaired *t*-tests and Bonferroni correction tests were conducted as appropriate using GraphPad Prism 10.6.1, comparing Cl-amidine-treated cells versus the respective comparative control group for each scenario, with significant differences reported as * *p* < 0.05, ** *p* < 0.01, *** *p* < 0.001 and **** *p* < 0.001.

## 5. Conclusions

In summary, this study highlights important roles for several PAD isozymes in CNS injury and regeneration, with possible differential, but also overlapping, roles of PAD isozymes in neuronal and astrocyte cells. Histone H3 citrullination was significantly reduced by pan-PAD inhibitor Cl-amidine in both cell types in most conditions tested, which correlated with increased wound-healing capacities in our in vitro acute CNS injury model of OGD/R. This highlights roles for histone H3 citrullination-mediated events, which can be linked to altered epigenetic regulation and NETosis. Findings, furthermore, highlight roles for several PAD isozymes, namely, PAD1, PAD2, PAD3 and PAD6 in neuronal cells, which warrants further exploration, while roles for PAD4 may be more dominant in astrocytes—albeit detection of PAD1, PAD2 and PAD3 was also identified. The findings support other studies on crucial roles for several PAD isozymes in CNS injury and open the platform for further in-depth assessments of PAD-isozyme-specific roles, as well as targeting a combination of PAD isozymes. The results support efforts in the neuroscience research field for development of PAD inhibitor strategies, using human in vitro models of neuronal injury and repair, to aid translation from bench to bedside.

## Data Availability

The original contributions presented in this study are included in the article/Supplementary Materials. Further inquiries can be directed to the corresponding author.

## Supplementary Materials

The following supporting information can be downloaded at: https://www.mdpi.com/article/10.3390/ijms27115118/s1.

## References

[1] Pilipović K., Harej Hrkać A., Kučić N., Mršić-Pelčić J. (2022). Modeling Central Nervous System Injury In Vitro: Current Status and Promising Future Strategies. Biomedicines.

[2] Acun C., Lavu R., Liu W., Nicoletti N., Ramsey J., Aly H. (2026). Therapeutic hypothermia in mild hypoxic ischemic encephalopathy: A clinical dilemma with uncertain long-term outcomes. Early Hum. Dev..

[3] Omelchenko A., Singh N.K., Firestein B.L. (2020). Current Advances in In Vitro Models of Central Nervous System Trauma. Curr. Opin. Biomed. Eng..

[4] Lange S., Gögel S., Leung K.-Y., Vernay B., Nicholas A.P., Causey C.P., Thompson P.R., Greene N.D.E., Ferretti P. (2011). Protein Deiminases: New Players in the Developmentally Regulated Loss of Neural Regenerative Ability. Dev. Biol..

[5] Lange S., Rocha-Ferreira E., Thei L., Mawjee P., Bennett K., Thompson P.R., Subramanian V., Nicholas A.P., Peebles D., Hristova M. (2014). Peptidylarginine Deiminases: Novel Drug Targets for Prevention of Neuronal Damage Following Hypoxic Ischemic Insult (HI) in Neonates. J. Neurochem..

[6] Lange S., Gallagher M., Kholia S., Kosgodage U.S., Hristova M., Hardy J., Inal J.M. (2017). Peptidylarginine Deiminases—Roles in Cancer and Neurodegeneration and Possible Avenues for Therapeutic Intervention via Modulation of Exosome and Microvesicle (EMV) Release?. Int. J. Mol. Sci..

[7] Lange S. (2016). Peptidylarginine Deiminases as Drug Targets in Neonatal Hypoxic-Ischemic Encephalopathy. Front. Neurol..

[8] Lange S. (2021). Peptidylarginine Deiminases and Extracellular Vesicles: Prospective Drug Targets and Biomarkers in Central Nervous System Diseases and Repair. Neural Regen. Res..

[9] Stadler S.C., Vincent C.T., Fedorov V.D., Patsialou A., Cherrington B.D., Wakshlag J.J., Mohanan S., Zee B.M., Zhang X., Garcia B.A. (2013). Dysregulation of PAD4-Mediated Citrullination of Nuclear GSK3β Activates TGF-β Signaling and Induces Epithelial-to-Mesenchymal Transition in Breast Cancer Cells. Proc. Natl. Acad. Sci. USA.

[10] Lazarus R.C., Buonora J.E., Flora M.N., Freedy J.G., Holstein G.R., Martinelli G.P., Jacobowitz D.M., Mueller G.P. (2015). Protein Citrullination: A Proposed Mechanism for Pathology in Traumatic Brain Injury. Front. Neurol..

[11] Attilio P.J., Flora M., Kamnaksh A., Bradshaw D.J., Agoston D.V., Mueller G.P. (2017). The Effects of Blast Exposure on Protein Deimination in the Brain. Oxidative Med. Cell. Longev..

[12] Beato M., Sharma P. (2020). Peptidyl Arginine Deiminase 2 (PADI2)-Mediated Arginine Citrullination Modulates Transcription in Cancer. Int. J. Mol. Sci..

[13] Boon L., Ugarte-Berzal E., Martens E., Fiten P., Vandooren J., Janssens R., Blanter M., Yu K., Boon M., Struyf S. (2021). Citrullination as a Novel Posttranslational Modification of Matrix Metalloproteinases. Matrix Biol..

[14] Sancandi M., Uysal-Onganer P., Kraev I., Mercer A., Lange S. (2020). Protein Deimination Signatures in Plasma and Plasma-EVs and Protein Deimination in the Brain Vasculature in a Rat Model of Pre-Motor Parkinson’s Disease. Int. J. Mol. Sci..

[15] Mercer A., Jaunmuktane Z., Hristova M., Lange S. (2022). Differential, Stage Dependent Detection of Peptidylarginine Deiminases and Protein Deimination in Lewy Body Diseases—Findings from a Pilot Study. Int. J. Mol. Sci..

[16] Mercer A., Sancandi M., MacLatchy A., Lange S. (2024). Brain-Region-Specific Differences in Protein Citrullination/Deimination in a Pre-Motor Parkinson’s Disease Rat Model. Int. J. Mol. Sci..

[17] Kosgodage U.S., Uysal-Onganer P., MacLatchy A., Kraev I., Chatterton N.P., Nicholas A.P., Inal J.M., Lange S. (2019). Peptidylarginine Deiminases Post-Translationally Deiminate Prohibitin and Modulate Extracellular Vesicle Release and MicroRNAs in Glioblastoma Multiforme. Int. J. Mol. Sci..

[18] Uysal-Onganer P., MacLatchy A., Mahmoud R., Kraev I., Thompson P.R., Inal J.M., Lange S. (2020). Peptidylarginine Deiminase Isozyme-Specific PAD2, PAD3 and PAD4 Inhibitors Differentially Modulate Extracellular Vesicle Signatures and Cell Invasion in Two Glioblastoma Multiforme Cell Lines. Int. J. Mol. Sci..

[19] Yusuf I.O., Parsi S., Ostrow L.W., Brown R.H., Thompson P.R., Xu Z. (2024). PAD2 Dysregulation and Aberrant Protein Citrullination Feature Prominently in Reactive Astrogliosis and Myelin Protein Aggregation in Sporadic ALS. Neurobiol. Dis..

[20] Liang H., Hunt J.B., Ma C., Kovalenko A., Calahatian J., Pedersen C., Liu H., Li J., Serrano M., Blazier D. (2025). Probing Tau Citrullination in Alzheimer’s Disease Brains and Mouse Models of Tauopathy. Acta Neuropathol..

[21] Bashir F., Awais H., Waseem A., Shahzad A., Khan A.B., Ali S.A., Shafiq L., Bhatti M.A. (2025). Structural and Mechanistic Insights into Peptidylarginine Deiminase (PAD2/PAD4)-Mediated Citrullination and Therapeutic Targeting: A Review. Int. J. Biol. Macromol..

[22] Dakin L.A., Xing L., Hall J., Ding W., Vajdos F.F., Pelker J.W., Ramsey S., Balbo P., Sahasrabudhe P.V., Banker M.E. (2025). Inhibiting Peptidylarginine Deiminases (PAD1-4) by Targeting a Ca^2+^-Dependent Allosteric Binding Site. Nat. Commun..

[23] Kijak-Boćkowska M., Czerwińska J., Owczarczyk-Saczonek A. (2025). Peptidylarginine Deiminases: An Overview of Recent Advances in Citrullination Research. Int. J. Mol. Sci..

[24] Arun P., Spadaro J., John J., Gharavi R.B., Bentley T.B., Nambiar M.P. (2011). Studies on Blast Traumatic Brain Injury Using In Vitro Model with Shock Tube. NeuroReport.

[25] Hatic H., Kane M.J., Saykally J.N., Citron B.A. (2012). Modulation of Transcription Factor Nrf2 in an In Vitro Model of Traumatic Brain Injury. J. Neurotrauma.

[26] Bae Y.-H., Joo H., Bae J., Hyeon S.J., Her S., Ko E., Choi H.G., Ryu H., Hur E.-M., Bu Y. (2018). Brain Injury Induces HIF-1α-Dependent Transcriptional Activation of LRRK2 That Exacerbates Brain Damage. Cell Death Dis..

[27] Chen W.C., Chang L.H., Huang S.S., Huang Y.J., Chih C.L., Kuo H.C., Lee Y.H., Lee I.H. (2019). Aryl Hydrocarbon Receptor Modulates Stroke-Induced Astrogliosis and Neurogenesis in the Adult Mouse Brain. J. Neuroinflamm..

[28] Jing Y., Yang D., Fu Y., Wang W., Yang G., Yuan F., Chen H., Ding J., Chen S., Tian H. (2019). Neuroprotective Effects of Serpina3k in Traumatic Brain Injury. Front. Neurol..

[29] Meyer L.J., Lotze F.P., Riess M.L. (2022). Simulated Traumatic Brain Injury in In Vitro Mouse Neuronal and Brain Endothelial Cell Culture Models. J. Pharmacol. Toxicol. Methods.

[30] Gallart-Palau X., Lee B.S.T., Adav S.S., Qian J., Serra A., Park J.E., Lai M.K.P., Chen C.P., Kalaria R.N., Sze S.K. (2016). Gender Differences in White Matter Pathology and Mitochondrial Dysfunction in Alzheimer’s Disease with Cerebrovascular Disease. Mol. Brain.

[31] Shi G., Liu L., Cao Y., Ma G., Zhu Y., Xu J., Zhang X., Li T., Mi L., Jia H. (2023). Inhibition of Neutrophil Extracellular Trap Formation Ameliorates Neuroinflammation and Neuronal Apoptosis via STING-Dependent IRE1α/ASK1/JNK Signaling Pathway in Mice with Traumatic Brain Injury. J. Neuroinflamm..

[32] Wu Y.H., Rosset S., Lee T.R., Dragunow M., Park T., Shim V. (2021). In Vitro Models of Traumatic Brain Injury: A Systematic Review. J. Neurotrauma.

[33] Chen K.-Z., Liu S.-X., Li Y.-W., He T., Zhao J., Wang T., Qiu X.-X., Wu H.-F. (2023). Vimentin as a Potential Target for Diverse Nervous System Diseases. Neural Regen. Res..

[34] Liu Q., Jin Z., Xu Z., Yang H., Li L., Li G., Li F., Gu S., Zong S., Zhou J. (2019). Antioxidant Effects of Ginkgolides and Bilobalide against Cerebral Ischemia Injury by Activating the Akt/Nrf2 Pathway In Vitro and In Vivo. Cell Stress Chaperones.

[35] Skrzypczak-Wiercioch A., Sałat K. (2022). Lipopolysaccharide-Induced Model of Neuroinflammation: Mechanisms of Action, Research Application and Future Directions for Its Use. Molecules.

[36] Ryou M.G., Mallet R.T. (2018). An In Vitro Oxygen-Glucose Deprivation Model for Studying Ischemia-Reperfusion Injury of Neuronal Cells. Trauma. Ischemic Inj. Methods Protoc..

[37] Babu M., Singh N., Datta A. (2022). In Vitro Oxygen Glucose Deprivation Model of Ischemic Stroke: A Proteomics-Driven Systems Biological Perspective. Mol. Neurobiol..

[38] Meixensberger J., Jaeger M., Väth A., Dings J., Kunze E., Roosen K. (2003). Brain tissue oxygen guided treatment supplementing ICP/CPP therapy after traumatic brain injury. J. Neurol. Neurosurg. Psychiatry.

[39] Luo Y., Arita K., Bhatia M., Knuckley B., Lee Y.-H., Stallcup M.R., Sato M., Thompson P.R. (2006). Inhibitors and Inactivators of Protein Arginine Deiminase 4: Functional and Structural Characterization. Biochemistry.

[40] Wang Y., Lyu Y., Tu K., Xu Q., Yang Y., Salman S., Le N., Lu H., Chen C., Zhu Y. (2021). Histone Citrullination by PADI4 Is Required for HIF-Dependent Transcriptional Responses to Hypoxia and Tumor Vascularization. Sci. Adv..

[41] Ahmed D., Puthussery H., Basnett P., Knowles J.C., Lange S., Roy I. (2021). Controlled Delivery of Pan-PAD-Inhibitor Cl-Amidine Using Poly(3-Hydroxybutyrate) Microspheres. Int. J. Mol. Sci..

[42] Muth A., Subramanian V., Beaumont E., Nagar M., Kerry P., McEwan P., Srinath H., Clancy K., Parelkar S., Thompson P.R. (2017). Development of a Selective Inhibitor of Protein Arginine Deiminase 2. J. Med. Chem..

[43] Willis V.C., Banda N.K., Cordova K.N., Chandra P.E., Robinson W.H., Cooper D.C., Lugo D., Mehta G., Taylor S., Tak P.P. (2017). Protein Arginine Deiminase 4 Inhibition Is Sufficient for the Amelioration of Collagen-Induced Arthritis. Clin. Exp. Immunol..

[44] Byun D.J., Lee J., Yu J.-W., Hyun Y.-M. (2023). NLRP3 Exacerbates NETosis-Associated Neuroinflammation in an LPS-Induced Inflamed Brain. Immune Netw..

[45] Frizzo J.K., Tramontina F., Bortoli E., Gottfried C., Leal R.B., Lengyel I., Donato R., Dunkley P.R., Gonçalves C.-A. (2004). S100B-Mediated Inhibition of the Phosphorylation of GFAP Is Prevented by TRTK-12. Neurochem. Res..

[46] Guerra M.C., Tortorelli L.S., Galland F., Da Ré C., Negri E., Engelke D.S., Rodrigues L., Leite M.C., Gonçalves C.-A. (2011). Lipopolysaccharide Modulates Astrocytic S100B Secretion: A Study in Cerebrospinal Fluid and Astrocyte Cultures from Rats. J. Neuroinflamm..

[47] Yang Z., Wang K.K. (2015). Glial Fibrillary Acidic Protein: From Intermediate Filament Assembly and Gliosis to Neurobiomarker. Trends Neurosci..

[48] Janigro D., Mondello S., Posti J.P., Unden J. (2022). GFAP and S100B: What You Always Wanted to Know and Never Dared to Ask. Front. Neurol..

[49] Richardson L.S., Emezienna N., Burd I., Taylor B.D., Peltier M.R., Han A., Menon R. (2022). Adapting an Organ-on-Chip Device to Study the Effect of Fetal Sex and Maternal Race/Ethnicity on Preterm Birth Related Intraamniotic Inflammation Leading to Fetal Neuroinflammation. Am. J. Reprod. Immunol..

[50] Cuende J., Moreno S., Bolaños J.P., Almeida A. (2008). Retinoic Acid Downregulates Rae1 Leading to APC^Cdh1^ Activation and Neuroblastoma SH-SY5Y Differentiation. Oncogene.

[51] Cheung Y.-T., Lau W.K.-W., Yu M.-S., Lai C.S.-W., Yeung S.-C., So K.-F., Chang R.C.-C. (2009). Effects of All-Trans-Retinoic Acid on Human SH-SY5Y Neuroblastoma as In Vitro Model in Neurotoxicity Research. Neurotoxicology.

[52] Xie H.-R., Hu L.-S., Li G.-Y. (2010). SH-SY5Y Human Neuroblastoma Cell Line: In Vitro Cell Model of Dopaminergic Neurons in Parkinson’s Disease. Chin. Med. J..

[53] Skotak M., Wang F., Chandra N. (2012). An In Vitro Injury Model for SH-SY5Y Neuroblastoma Cells: Effect of Strain and Strain Rate. J. Neurosci. Methods.

[54] Filograna R., Civiero L., Ferrari V., Codolo G., Greggio E., Bubacco L., Beltramini M., Bisaglia M. (2015). Analysis of the Catecholaminergic Phenotype in Human SH-SY5Y and BE(2)-M17 Neuroblastoma Cell Lines upon Differentiation. PLoS ONE.

[55] Elnagar M.R., Walls A.B., Helal G.K., Hamada F.M., Thomsen M.S., Jensen A.A. (2018). Functional Characterization of α7 Nicotinic Acetylcholine and NMDA Receptor Signaling in SH-SY5Y Neuroblastoma Cells in an ERK Phosphorylation Assay. Eur. J. Pharmacol..

[56] Juntunen M., Hagman S., Moisan A., Narkilahti S., Miettinen S. (2020). In Vitro Oxygen-Glucose Deprivation-Induced Stroke Models with Human Neuroblastoma Cell- and Induced Pluripotent Stem Cell-Derived Neurons. Stem Cells Int..

[57] Wan F., Jin L., Qin Y., Zeng Y. (2023). Modulation of Muscarinic Receptors by Anisodine Hydrobromide in Cerebral Ischemia. Cell. Mol. Biol..

[58] Thakor F.K., Wan K.-W., Welsby P.J., Welsby G. (2017). Pharmacological Effects of Asiatic Acid in Glioblastoma Cells under Hypoxia. Mol. Cell. Biochem..

[59] Demircan T., Yavuz M., Kaya E., Akgül S., Altuntaş E. (2021). Cellular and Molecular Comparison of Glioblastoma Multiform Cell Lines. Cureus.

[60] Cao Y., Shi M., Liu L., Zuo Y., Jia H., Min X., Liu X., Chen Z., Zhou Y., Li S. (2023). Inhibition of Neutrophil Extracellular Trap Formation Attenuates NLRP1-Dependent Neuronal Pyroptosis via STING/IRE1α Pathway after Traumatic Brain Injury in Mice. Front. Immunol..

[61] Zhang T., Liu P., Yao G., Zhang X., Cao C. (2023). A complex heterozygous mutation in *PADI6* causes early embryo arrest: A case report. Front Genet..

[62] Williams J.P.C., Mouilleron S., Trapero R.H., Bertran M.T., Marsh J.A., Walport L.J. (2024). Structural Insight into the Function of Human Peptidyl Arginine Deiminase 6. Comput. Struct. Biotechnol. J..

[63] Zhu Y., Xu J., Chai Y., Li P., Liu L., Zhang S., Zhang J., Chen X. (2025). Neutrophil Extracellular Traps Aggravate Blood-Brain Barrier Disruption via ZBP1/FSP1-Mediated Ferroptosis after Traumatic Brain Injury. Fluids Barriers CNS.

[64] Lai N.-S., Yu H.-C., Tung C.-H., Huang K.-Y., Huang H.-B., Lu M.-C. (2019). Increased Peptidylarginine Deiminases Expression during the Macrophage Differentiation and Participated Inflammatory Responses. Arthritis Res. Ther..

[65] Chen Y., Zhang H., Hu X., Cai W., Ni W., Zhou K. (2022). Role of NETosis in Central Nervous System Injury. Oxidative Med. Cell. Longev..

[66] Savi M., Su F., Sterchele E.D., Bogossian E.G., Demailly Z., Baggiani M., Casu G.S., Taccone F.S. (2024). Targeting NETosis in Acute Brain Injury: A Systematic Review of Preclinical and Clinical Evidence. Cells.

[67] Qiao S., Yuan J., Zhang S.-C., Lu Y.-Y., Zhou P., Xin T. (2025). Neutrophil Extracellular Traps in Central Nervous System Disorders: Mechanisms, Implications, and Emerging Perspective. Front. Immunol..

[68] Seol S.-I., Oh S.-A., Davaanyam D., Lee J.-K. (2025). Blocking Peptidyl Arginine Deiminase 4 Confers Neuroprotective Effect in the Post-Ischemic Brain through Both NETosis-Dependent and -Independent Mechanisms. Acta Neuropathol. Commun..

[69] Zhou R., Zhang T., Sun J., Tan M., Li T., Yang T., Dai S.-S., Liu Y.-W. (2025). Neutrophil Extracellular Traps Aggravate Neutrophil Reverse Transendothelial Migration during Traumatic Brain Injury. Biochem. Biophys. Res. Commun..

[70] Li A., Pei T.W., Qi H., Song L.B., Fang J., Ding Z.S., Chen T. (2026). Study on the Function and Mechanism of Neutrophil Extracellular Traps in Regulating Necroptosis Following Traumatic Brain Injury. Brain Behav..

[71] Wood L.M., Moore J.K. (2025). β3 Accelerates Microtubule Plus End Maturation through a Divergent Lateral Interface. Mol. Biol. Cell.

[72] Poirier K., Saillour Y., Bahi-Buisson N., Jaglin X.H., Fallet-Bianco C., Nabbout R., Castelnau-Ptakhine L., Roubertie A., Attié-Bitach T., Desguerre I. (2010). Mutations in the Neuronal β-Tubulin Subunit TUBB3 Result in Malformation of Cortical Development and Neuronal Migration Defects. Hum. Mol. Genet..

[73] Kaverina I., Straube A. (2011). Regulation of Cell Migration by Dynamic Microtubules. Semin. Cell Dev. Biol..

[74] Quigley E.B., DeVore S.B., Khan S.A., Geisterfer Z.M., Rothfuss H.M., Sequoia A.O., Thompson P.R., Gatlin J.C., Cherrington B.D., Navratil A.M. (2024). GnRH Induces Citrullination of the Cytoskeleton in Murine Gonadotrope Cells. Int. J. Mol. Sci..

[75] Moskowitz P.F., Oblinger M.M. (1995). Sensory Neurons Selectively Upregulate Synthesis and Transport of the Beta III-Tubulin Protein during Axonal Regeneration. J. Neurosci..

[76] Latremoliere A., Cheng L., DeLisle M., Wu C., Chew S., Hutchinson E.B., Sheridan A., Alexandre C., Latremoliere F., Sheu S.-H. (2018). Neuronal-Specific TUBB3 Is Not Required for Normal Neuronal Function but Is Essential for Timely Axon Regeneration. Cell Rep..

[77] Puri D., Barry B.J., Engle E.C. (2023). TUBB3 and KIF21A in Neurodevelopment and Disease. Front. Neurosci..

[78] Guo Y., Wang Y.-Y., Sun T.-T., Xu J.-J., Yang P., Ma C.-Y., Guan W.-J., Wang C.-J., Liu G.-F., Liu C.-Q. (2023). Neural Progenitor Cells Derived from Fibroblasts Induced by Small Molecule Compounds under Hypoxia for Treatment of Parkinson’s Disease in Rats. Neural Regen. Res..

[79] Hartmann J., Henschel N., Bartmann K., Dönmez A., Brockerhoff G., Koch K., Fritsche E. (2023). Molecular and Functional Characterization of Different BrainSphere Models for Use in Neurotoxicity Testing on Microelectrode Arrays. Cells.

[80] Kim J.-T., Cho S.M., Youn D.H., Hong E.P., Park C.H., Lee Y., Jung H., Jeon J.P. (2023). Therapeutic Effect of a Hydrogel-Based Neural Stem Cell Delivery Sheet for Mild Traumatic Brain Injury. Acta Biomater..

[81] Wang G., Wang W., Zhang Y., Gou X., Zhang Q., Huang Y., Zhang K., Zhang H., Yang J., Li Y. (2024). Ethanol Changes Nestin-Promoter Induced Neural Stem Cells to Disturb Newborn Dendritic Spine Remodeling in the Hippocampus of Mice. Neural Regen. Res..

[82] Wilhelmsson U., Lebkuechner I., Leke R., Marasek P., Yang X., Antfolk D., Chen M., Mohseni P., Lasič E., Bobnar S.T. (2019). Nestin Regulates Neurogenesis in Mice through Notch Signaling from Astrocytes to Neural Stem Cells. Cereb. Cortex.

[83] Kee N., Sivalingam S., Boonstra R., Wojtowicz J.M. (2002). The utility of Ki-67 and BrdU as proliferative markers of adult neurogenesis. J. Neurosci. Methods.

[84] Gilyarov A.V. (2008). Nestin in Central Nervous System Cells. Neurosci. Behav. Physiol..

[85] Xiao Q.-X., Xue L.-L., Tan Y.-X., Huangfu L.-R., Chen L., Zhai C.-Y., Ma R.-F., Al-Hawwas M., Zhou H.-S., Wang T.-H. (2024). p75ECD-Fc reverses neonatal hypoxic-ischemic encephalopathy-induced neurological deficits and inhibits apoptosis associated with Nestin. Biomed. Pharmacother..

[86] Nishie H., Nakano-Doi A., Sawano T., Nakagomi T. (2021). Establishment of a Reproducible Ischemic Stroke Model in Nestin-GFP Mice with High Survival Rates. Int. J. Mol. Sci..

[87] Ramaswamy S., Goings G.E., Soderstrom K.E., Szele F.G., Kozlowski D.A. (2005). Cellular proliferation and migration following a controlled cortical impact in the mouse. Brain Res..

[88] Briot J., Simon M., Méchin M.-C. (2020). Deimination, Intermediate Filaments and Associated Proteins. Int. J. Mol. Sci..

[89] Mafuika N.S., Naicker T., Harrichandparsad R., Lazarus L. (2022). The Potential of Serum S100 Calcium-Binding Protein B and Glial Fibrillary Acidic Protein as Biomarkers for Traumatic Brain Injury. Transl. Res. Anat..

[90] Vos P.E., Jacobs B., Andriessen T.M.J.C., Lamers K.J.B., Borm G.F., Beems T., Edwards M., Rosmalen C.F., Vissers J.L.M. (2010). GFAP and S100B Are Biomarkers of Traumatic Brain Injury: An Observational Cohort Study. Neurology.

[91] Agoston D.V., Shutes-David A., Peskind E.R. (2017). Biofluid Biomarkers of Traumatic Brain Injury. Brain Inj..

[92] Czeiter E., Amrein K., Gravesteijn B.Y., Lecky F., Menon D.K., Mondello S., Newcombe V.F.J., Richter S., Steyerberg E.W., Vyvere T.V. (2020). Blood Biomarkers on Admission in Acute Traumatic Brain Injury: Relations to Severity, CT Findings and Care Path in the CENTER-TBI Study. EBioMedicine.

[93] Wizeman J.W., Nicholas A.P., Ishigami A., Mohan R. (2016). Citrullination of glial intermediate filaments is an early response in retinal injury. Mol. Vis..

[94] Yousaf M.A., Scartezzini A., Colombo C., Bachetti T., Sarto E., Bella D.D., Lorenzi P., Tinazzi M., Fabrizi G.M., Vattemi G. (2025). A Novel De Novo GFAP Variant Causes a Juvenile-Onset Alexander Disease with Bilateral Vocal Cord Paralysis. Gene.

[95] Rodnight R., Gonçalves C.A., Wofchuk S.T., Leal R. (1997). Control of the Phosphorylation of the Astrocyte Marker Glial Fibrillary Acidic Protein (GFAP) in the Immature Rat Hippocampus by Glutamate and Calcium Ions: Possible Key Factor in Astrocytic Plasticity. Braz. J. Med. Biol. Res..

[96] Herskowitz J.H., Seyfried N.T., Duong D.M., Xia Q., Rees H.D., Gearing M., Peng J., Lah J.J., Levey A.I. (2010). Phosphoproteomic Analysis Reveals Site-Specific Changes in GFAP and NDRG2 Phosphorylation in Frontotemporal Lobar Degeneration. J. Proteome Res..

[97] Sullivan S.M., Sullivan R.K.P., Miller S.M., Ireland Z., Björkman S.T., Pow D.V., Colditz P.B. (2012). Phosphorylation of GFAP Is Associated with Injury in the Neonatal Pig Hypoxic-Ischemic Brain. Neurochem. Res..

[98] Snider N.T., Omary M.B. (2014). Post-Translational Modifications of Intermediate Filament Proteins: Mechanisms and Functions. Nat. Rev. Mol. Cell Biol..

[99] Battaglia R.A., Beltran A.S., Delic S., Dumitru R., Robinson J.A., Kabiraj P., Herring L.E., Madden V.J., Ravinder N., Willems E. (2019). Site-Specific Phosphorylation and Caspase Cleavage of GFAP Are New Markers of Alexander Disease Severity. eLife.

[100] Kanuri S.H., Sirrkay P.J. (2025). Deciphering the Structural Biology of GFAP: Connotations of Its Potency in Presaging the Diagnosis for Traumatic Brain Injury and AD. Neurol. Int..

[101] Klein M.A., Möller J.C., Jones L.L., Bluethmann H., Kreutzberg G.W., Raivich G. (1997). Impaired Neuroglial Activation in Interleukin-6 Deficient Mice. Glia.

[102] Brunello A.G., Weissenberger J., Kappeler A., Vallan C., Peters M., Rose-John S., Weis J. (2000). Astrocytic Alterations in Interleukin-6/Soluble Interleukin-6 Receptor Alpha Double-Transgenic Mice. Am. J. Pathol..

[103] Levison S.W., Jiang F.J., Stoltzfus O.K., Ducceschi M.H. (2000). IL-6-Type Cytokines Enhance Epidermal Growth Factor-Stimulated Astrocyte Proliferation. Glia.

[104] Krasovska V., Doering L.C. (2018). Regulation of IL-6 Secretion by Astrocytes via TLR4 in the Fragile X Mouse Model. Front. Mol. Neurosci..

[105] Pons-Espinal M., Blasco-Agell L., Fernandez-Carasa I., Andrés-Benito P., di Domenico A., Richaud-Patin Y., Baruffi V., Marruecos L., Espinosa L., Garrido A. (2024). Blocking IL-6 Signaling Prevents Astrocyte-Induced Neurodegeneration in an iPSC-Based Model of Parkinson’s Disease. JCI Insight.

[106] Ishigami A., Ohsawa T., Hiratsuka M., Taguchi H., Kobayashi S., Saito Y., Murayama S., Asaga H., Toda T., Kimura N. (2005). Abnormal Accumulation of Citrullinated Proteins Catalyzed by Peptidylarginine Deiminase in Hippocampal Extracts from Patients with Alzheimer’s Disease. J. Neurosci. Res..

[107] Jang B., Jin J.-K., Jeon Y.-C., Cho H.J., Ishigami A., Choi K.-C., Carp R.I., Maruyama N., Kim Y.-S., Choi E.-K. (2010). Involvement of Peptidylarginine Deiminase-Mediated Post-Translational Citrullination in Pathogenesis of Sporadic Creutzfeldt-Jakob Disease. Acta Neuropathol..

[108] Yusuf I.O., Qiao T., Parsi S., Tilvawala R., Thompson P.R., Xu Z. (2022). Protein Citrullination Marks Myelin Protein Aggregation and Disease Progression in Mouse ALS Models. Acta Neuropathol. Commun..

[109] McNee G., Eales K.L., Wei W., Williams D.S., Barkhuizen A., Bartlett D.B., Essex S., Anandram S., Filer A., Moss P.A.H. (2017). Citrullination of Histone H3 Drives IL-6 Production by Bone Marrow Mesenchymal Stem Cells in MGUS and Multiple Myeloma. Leukemia.

[110] Hillman T.C., Jacobson B., De Medoza K.P.H., Lopez M., Iwakoshi N., Wilson C.G. (2025). Inflammation mediated brain damage and cytokine expression in a maternally derived murine model for preterm hypoxic-ischemic encephalopathy. Front. Syst. Biol..

[111] Abikenari M., Ha J.H., Liu J., Ren A., Cho K.B., Lim J., Kim L.H., Medikonda R., Choi J., Lim M. (2025). The immunological landscape of traumatic brain injury: Insights from pathophysiology to experimental models. Front. Neurol..

[112] Huo Q., Zhang Y., Zhao J., Bai N., Wang J. (2026). HIF-1α/BNIP3-mediated mitophagy mitigates cerebral ischemia/reperfusion injury in rats by suppressing NLRP3 inflammasome activation. Eur. J. Med. Res..

[113] Puthusseri S.P., Ravivarma S., Johny M., Vengellur A. (2026). Hypoxia-inducible factor-1α: Dual roles in maintaining neuronal homeostasis and neuronal degeneration via regulation of oxidative stress, mitochondrial dynamics, and bioenergetics. J. Physiol. Biochem..

[114] De Conto V., Cheung V., Maubon G., Souguir Z., Maubon N., Vandenhaute E., Bérézowski V. (2021). In Vitro Differentiation Modifies the Neurotoxic Response of SH-SY5Y Cells. Toxicol. In Vitr..

[115] U K.P., Subramanian V., Nicholas A.P., Thompson P.R., Ferretti P. (2014). Modulation of Calcium-Induced Cell Death in Human Neural Stem Cells by the Novel Peptidylarginine Deiminase-AIF Pathway. Biochim. Biophys. Acta BBA Mol. Cell Res..

[116] Kaneva M.K., Kerrigan M.J., Grieco P., Curley G.P., Locke I.C., Getting S.J. (2012). Chondroprotective and Anti-Inflammatory Role of Melanocortin Peptides in TNF-α Activated Human C-20/A4 Chondrocytes. Br. J. Pharmacol..

[117] Can V.C., Locke I.C., Kaneva M.K., Kerrigan M.J.P., Merlino F., De Pascale C., Grieco P., Getting S.J. (2020). Novel Anti-Inflammatory and Chondroprotective Effects of the Human Melanocortin MC1 Receptor Agonist BMS-470539 Dihydrochloride and Human Melanocortin MC3 Receptor Agonist PG-990 on Lipopolysaccharide Activated Chondrocytes. Eur. J. Pharmacol..

