# Supplementary material for: Targeting Peptidylarginine Deiminases in Neurons and Astrocytes in Central Nervous System Injury—Effects of Pan-PAD Inhibitor Cl-Amidine in an Oxygen–Glucose Deprivation Model of Ischaemia (OGD/R) and LPS Stimulation In Vitro
Source: Int J Mol Sci. 2026 Jun 5;27(11):5118. doi: 10.3390/ijms27115118 (PMC13257080; doi:10.3390/ijms27115118)
Supplement: Supplementary file 1 [file ijms-27-05118-s001.zip › ijms-4296338-supplementary.pdf]

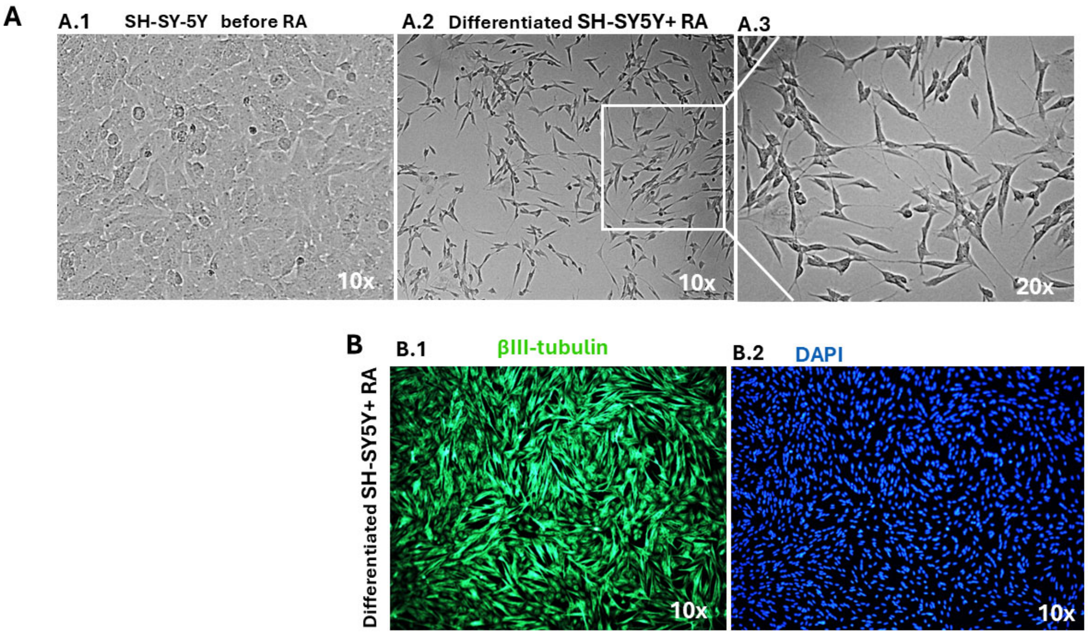

Figure S1: Differentiated SH-SY5Y cells show positive for βIII-tubulin **A**. Brightfield shows undifferentiated (A.1) compared with differentiated (A2.-A-3) SH-SY5Y cells (following 6 day differentiation with retinoic acid, RA) captured using the 10x and 20x objective; clear neuronal morphology is observed for differentiated SH-SY5Y cells (A2, A3). **B.** Immunocytochemical staining of the differentiated SH-SY5Y cells showing the positive βIII-tubulin staining as a marker of mature neurons (B.1) and nuclear DAPI staining (B.2),. All figures were visualized using the EVOS_FL2 system.
